# Geochemically Defined Space-for-Time Transects Successfully Capture Microbial Dynamics Along Lacustrine Chronosequences in a Polar Desert

**DOI:** 10.3389/fmicb.2021.783767

**Published:** 2022-01-31

**Authors:** Maria R. Monteiro, Alexis J. Marshall, Ian Hawes, Charles K. Lee, Ian R. McDonald, Stephen Craig Cary

**Affiliations:** ^1^International Centre for Terrestrial Antarctic Research, University of Waikato, Hamilton, New Zealand; ^2^Te Aka Matuatua—School of Science, University of Waikato, Hamilton, New Zealand

**Keywords:** space-for-time (SFT) substitution, climate change, polar desert environments, microbial communities, wetness gradients, Antarctica

## Abstract

The space-for-time substitution approach provides a valuable empirical assessment to infer temporal effects of disturbance from spatial gradients. Applied to predict the response of different ecosystems under current climate change scenarios, it remains poorly tested in microbial ecology studies, partly due to the trophic complexity of the ecosystems typically studied. The McMurdo Dry Valleys (MDV) of Antarctica represent a trophically simple polar desert projected to experience drastic changes in water availability under current climate change scenarios. We used this ideal model system to develop and validate a microbial space-for-time sampling approach, using the variation of geochemical profiles that follow alterations in water availability and reflect past changes in the system. Our framework measured soil electrical conductivity, pH, and water activity *in situ* to geochemically define 17 space-for-time transects from the shores of four dynamic and two static Dry Valley lakes. We identified microbial taxa that are consistently responsive to changes in wetness in the soils and reliably associated with long-term dry or wet edaphic conditions. Comparisons between transects defined at static (open-basin) and dynamic (closed-basin) lakes highlighted the capacity for geochemically defined space-for-time gradients to identify lasting deterministic impacts of historical changes in water presence on the structure and diversity of extant microbial communities. We highlight the potential for geochemically defined space-for-time transects to resolve legacy impacts of environmental change when used in conjunction with static and dynamic scenarios, and to inform future environmental scenarios through changes in the microbial community structure, composition, and diversity.

## Introduction

Long-term ecological observations provide valuable information for studying the impacts of climate change. They form excellent resources to detect climate trends and patterns over time, study slow or highly variable ecological processes, validate modeled predictions of change, and support environmental policies (Kratz et al., 2003; Rustad, 2008). However, the maintenance of these continuous observations is generally dependent on long-term financial and logistic security from local institutions and governments. When time and funding are a constraint, or when long-term studies are not feasible, space-for-time substitution models are an attractive alternative to forecast long-term climate impacts on ecosystems (Blois et al., 2013).

Space-for-time substitution approaches, such as ecological chronosequences, rely on the assumption that factors responsible for spatial turnover in species abundance are similar to those responsible for temporal turnover (Pickett, 1989; Wogan and Wang, 2018). First used to study plant succession and soil development (Pickett, 1989; Johnson and Miyanishi, 2008; Walker et al., 2010), it has recently been adapted to predict impacts of climate change on microbial communities (Wilhelm et al., 2013; Yang et al., 2014; Yan et al., 2017; Colby et al., 2020). However, despite its common use, the reliability of this approach has been questioned, particularly when its primary assumption is not met or tested (Blois et al., 2013; Damgaard, 2019). For instance, deterministic and stochastic processes can both affect how different microbial groups assemble over different space and time scales (Caruso et al., 2011). Therefore, space-for-time sampling designs should consider the scale and history of the sampling site and discuss the legacy impacts left by historical processes that have known lasting effects on microbial communities (Chase and Myers, 2011; Dini-Andreote et al., 2015; Zhou and Ning, 2017). Without *a priori* knowledge of the ecological niche characteristics or empirical measurements that constrain the deterministic drivers of species variation, space-for-time sampling approaches may lead to a naive interpretation of the community, missing spatial/temporal context, and impairing any comparisons between replicated spatial transects. Ideally, to validate the use of this approach to assess the temporal effects of climate change on an ecosystem’s microbiome, a baseline study is required in an ecosystem that lacks trophic complexity and includes well-characterized deterministic gradients of species distribution.

The McMurdo Dry Valleys (MDV) are the largest ice-free area in Antarctica and represent one of Earth’s coldest and driest regions (Cary et al., 2010). These polar deserts exhibit high spatial variability in geochemistry, climate, and landscape characteristics, resulting in a patchy distribution of a simple and unique biota functionally dominated by microbial communities (Cary et al., 2010; Lee et al., 2012; Kwon et al., 2017; Feeser et al., 2018; Bottos et al., 2020). Since the environmental conditions select against the establishment of vascular plants and limit complex trophic interactions, the MDV represents an ideal natural laboratory to validate space-for-time as a tool to study climate-related disturbances on microbial communities. Recent changes in the local climate have triggered hydrologic responses across the MDV (Castendyk et al., 2016; Fountain et al., 2016; Levy et al., 2018). This is especially relevant in closed-basin lakes, which are described as lakes that do not have an outlet channel for water to flow out. Examples of these type of lakes include Lake Vanda, Lake Bonney, and Lake Fryxell in the Taylor and Wright Valleys, where the water level has risen (Castendyk et al., 2016; Levy et al., 2018). Water level rise triggers the expansion of the adjacent wetted margins, which imposes selective pressures on the established microbial communities adapted to long-term dry conditions (Van Horn et al., 2014; Niederberger et al., 2015, 2019; Buelow et al., 2016; Lee et al., 2018; Coyne et al., 2020; Ramoneda Massague et al., 2021). Our work and others have previously used simplistic sampling approaches based on physical distance to better understand how microbial community diversity responds to geochemical changes along an environmental gradient (Yang et al., 2014; Niederberger et al., 2015; Yan et al., 2017; Feeser et al., 2018; Lee et al., 2018). These studies assume that geochemical variables along a gradient change gradually and linearly with distance. However, interacting environmental factors may not continuously change along a distance-based gradient (Kappes et al., 2010), nor are microbial communities randomly distributed along natural gradients, being continuously under the influence of deterministic, stochastic, or a combination of both processes.

In this study, our goal was to develop and validate a space-for-time sampling approach to assess the impacts of climate-related hydrological changes on the terrestrial microbiome in a polar desert, using a wetness gradient. We achieved this by determining whether extant microbial community attributes (e.g., changes in structure and diversity) across replicated geochemically defined space-for-time transects could reconstruct past wetting events within the MDV. To ensure the robustness of the transects, we first methodically characterized the spatial variability of local geochemical parameters (water activity, electrical conductivity, and pH) at the chosen sites. Transects were established across static (open-basin with outflow) and dynamic (closed-basin with no outflow) lakes to identify if the structure, diversity, and composition of the microbial community could be used to assess historic vs. more recent impacts of water availability. Open-basin lakes are expected to be less impacted by increased glacial meltwater and groundwater flow due to the presence of an outflow channel which drains the overflow. Therefore, wetted areas surrounding these lakes are expected to expand at lower rates and be more stable, comparatively to those surrounding closed-basin lakes which reflect more recent and predicted hydrological disturbances in the system (Castendyk et al., 2016). We demonstrate the strength of extant microbial community attributes for reconstructing past impacts of hydrological changes and support the capacity for space-for-time to be developed as a robust tool to understand future impacts of change under current climate warming scenarios.

## Materials and Methods

### Site Description, Space-for-Time Transects, and Sampling

**FIGURE 1:**
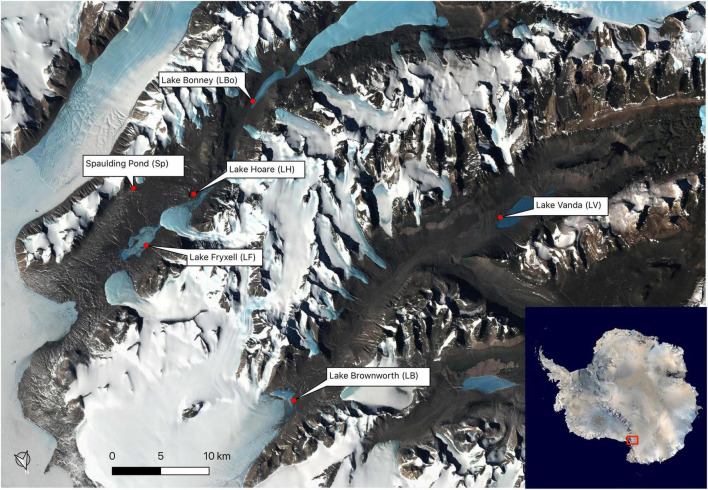
Sampling locations in Wright Valley and Taylor Valley.

We geochemically defined seventeen space-for-time soil transects, representing a wetness gradient from the shores of six lakes during the 2016 and 2017 Antarctic field seasons. The lakes span the length of the Wright (Lake Brownworth and Lake Vanda) and Taylor (Spaulding Pond, Lake Fryxell, Lake Hoare, and Lake Bonney) valleys (Figure 1 and Table 1). Lake Brownworth and Spaulding Pond are open-basin lakes, meaning that they have an outlet channel through which water flows out. The presence of the outlet regulates the water level in these lakes (Levy et al., 2018). Transects defined from these lakes are referred to in this study as static (as having more static water levels). Lakes Vanda, Fryxell, Bonney, and Hoare are closed-basin lakes, which do not have an outlet channel for water to flow out of the lake. The water levels in these lakes are dynamic on multiple time scales but, over the last 100 years, are thought to have been intermittently rising (Castendyk et al., 2016; Levy et al., 2018; Ramoneda Massague et al., 2021). Transects defined from these lakes are referred to in this study as dynamic (as having more dynamic water levels). For each lake, except for Lake Hoare, three space-for-time transects were defined along the wetness gradient. At Lake Hoare, only two transects were sampled due to snowfall while sampling.

**TABLE 1:** Soil samples collected across all space-for-time transects and associated geochemical data.

Lake	Lat and long	Valley	Transect section	Gradient	Sampling point	Elevation (cm)	Distance from shoreline (m)	EC (μS)	WA	pH	Moisture (%)
Lake brownworth (LB)	S77.42414 E162.73761	Wright	Wet	Static	1	0	0	25.45 (10.9)	1	7.3 (0.42)	16.77 (3.28)
			Transition		2	14.33 (1.5)	5 (3.6)	71.7 (36.6)	1	7.8 (0.11)	11.30 (1.58)
			Transition		3	36.13 (3.53)	7.35 (3.44)	321 (194.7)	0.77 (0.25)	7.89 (0.10)	6.40 (2.90)
			Dry		4	45.73 (1.41)	8.25 (3.27)	205.7 (87.2)	0.43 (0.11)	7.87 (0.16)	0.74 (0.46)
			Dry		5	92.17 (6.17)	13.7 (3.05)	61.2 (54.5)	0.33 (0.05)	7.87 (0.32)	0.35 (0.6)
Lake vanda (LV)	S77.53408 E161.62372	Wright	Wet	Dynamic	1	0	0	160 (42.23)	1	8.06 (0.11)	18.65 (1.58)
			Transition		2	15.8 (3.34)	2.3 (0.58)	796.8 (154)	0.93 (0.11)	7.9 (0.10)	8.74 (2.9)
			Transition		3	35 (3.37)	3.7 (0.64)	2,340 (2,303)	0.46 (0.38)	7.63 (0.11)	1.06 (0.55)
			Dry		4	45.63 (9.27)	4.83 (1.04)	156.2 (155)	0.26 (0.06)	7.57 (0.15)	0.14 (0.12)
			Dry		5	138.85 (6.15)	11 (1.41)	72.1 (40.44)	0.25 (0.07)	7.45 (0.07)	0.11 (0.14)
Lake bonney (LBo)	S77.69933 E162.53149	Taylor	Wet	Dynamic	1	0	0	83.1 (19.1)	1	8 (0.24)	17.07 (0.28)
			Transition		2	9.83 (0.29)	1	705.7 (302.2)	1	8.44 (0.41)	11.30 (17.63)
			Transition		3	84.67 (2.57)	5.97 (0.05)	5,300 (1,503)	0.31 (0.02)	8.10 (0.15)	2.06 (0.6)
			Dry		4	105 (8.41)	7.5 (0.87)	1269.77 (344.24)	0.40 (0.26)	8.19 (0.07)	1.75 (0.94)
			Dry		5	116.25 (2.48)	8	1217.9 (712.90)	0.28 (0.05)	8.25 (0.13)	0.63 (0.40)
Lake hoare (LH)	S77.63271 E162.93942	Taylor	Wet	Dynamic	1	0	0	160 (61.05)	1	8.47 (0.03)	11.93 (0.51)
			Transition		2	13.25 (1.06)	1	788.25 (761.90)	1	8.76 (0.27)	8.60 (2.58)
			Transition		3	43.75 (1.06)	2.5 (0.7)	475.85 (136.11)	0.46 (0.64)	9.05 (0.16)	2.94 (1.16)
			Dry		4	57.5 (0.70)	3 (0.70)	267.4 (154)	0.6	8.75 (0.30)	1.19 (0.12)
			Dry		5	136 (14.85)	7	133.6 (31.96)	0.8	8.75 (0.20)	0.42 (0.35)
Lake fryxell (LF)	S77.60336 E163.13921	Taylor	Wet	Dynamic	1	0	0	159 (82.53)	1	8.22 (0.12)	16.90 (1.59)
			Transition		2	6 (4.58)	1	633.20 (275.07)	0.98 (0.04)	8.25 (0.30)	12.82 (3.58)
			Transition		3	52.17 (2.57)	7.67 (1.15)	1582.33 (302)	0.98 (0.01)	8.20 (0.02)	6.45 (2.38)
			Dry		4	64.33 (2.52)	8.83 (1.04)	106.26 (34.93)	1 (0.05)	8.47 (0.03)	1.86 (0.53)
			Dry		5	161 (0.12)	19.83 (0.76)	161.12 (99.83)	1 (0.02)	8.69 (0.20)	1.42 (0.37)
Spaulding pond (Sp)	S77.65936 E163.12224	Taylor	Wet	Static	1	0	0	210.63 (107.97)	1	7.69 (0.24)	20.63 (4.44)
			Transition		2	14.83 (2.47)	1.83 (1.04)	765.63 (403.19)	1	8.71 (0.25)	11.87 (2.52)
			Transition		3	27.17 (8.46)	3.67 (1.15)	469.17 (304.77)	1	8.69 (0.19)	4.85 (3.07)
			Dry		4	68.83 (3.25)	7 (2.65)	198.60 (88.51)	0.78 (0.14)	8.53 (0.11)	0.40 (0.16)
			Dry		5	122.33 (20.14)	14.67 (1.15)	355.47 (231.73)	0.88 (0.16)	8.44 (0.27)	0.56 (0.10)

*Values represent the average and standard deviations of the measurements across replicated transects (n = 3), except for Lake Hoare (n = 2). Elevation from the shoreline (cm), distance from the shoreline (m), electrical conductivity (EC) (μS), water activity (WA), pH, and soil moisture (%).*

In these soils, pH, electrical conductivity (EC) and water activity (WA) have been described in several studies as being the key drivers of community assembly in the MDV (Barrett et al., 2009; Van Horn et al., 2014; Niederberger et al., 2015, 2019; Feeser et al., 2018; Bottos et al., 2020; George et al., 2021). These metrics were measured in the field to identify *in situ* geochemical gradients across transects from all water bodies within which three geochemical zones were identified (Table 1 and Supplementary Figures 1, 2). This process involved performing measurements with an average spacing of 50 cm from the existing water level within a transition zone to observe the natural variation of these geochemical variables along the wetness gradient (Supplementary Figures 1, 2). The first zone represented a water-saturated wet zone characterized by comparatively higher water activity (average of 1) and the lowest electrical conductivity (138 ± 81 μS) (sampling point 1). With increasing distance from the lake shore, water content remained high, but conductivity increased several times (938 ± 1,591 μS) (sampling points 2 and 3). This was used to define the second zone, representative of a transition zone. The third zone represented a dry zone, characterized by the lowest measured water activity and lower electrical conductivity (341 ± 443 μS) (sampling points 4 and 5).

Briefly, electrical conductivity and pH were determined from a 1:5 slurry of soil to Milli-Q water (Thermo Scientific Orion meter, United States) (Lee et al., 2012). Water activity was measured using a PawKit meter (AquaLab, NZ), following the manufacturer’s instructions. Infield measurements of electrical conductivity and pH were confirmed under laboratory conditions using Thermo Scientific Orion meter and the same slurry technique, but with the addition of 0.5 mL of 0.01M CaCl_2_ per sample (Minasny et al., 2011; Lee et al., 2012). Soil moisture content was determined according to Lee et al. (2012). The elevation of each sampling point along the transects was surveyed in relation to the lakeshore (sampling point 1) using a laser leveling system (Topcon Laser Systems, United States).

For microbial community analysis, we aseptically collected surface soil samples (top 2 cm) with a sterile spatula from the five geochemically predefined sampling points. Spatulas were washed and then sterilized with ethanol wipes between sampling points and sampling sites. In total, across the six lakes, seventeen space-for-time transects were sampled, resulting in 85 soil samples. Individual samples were thoroughly mixed in a sterile Whirl-Pak^®^ bag and then distributed into sterile 50 mL Falcon tubes. Samples were initially stored in the field on ice for 24 h before being transferred to dry ice and transported to Scott Base (the New Zealand Antarctic Research Station), where they were maintained a –60°C until DNA extraction. The remaining samples in each Whirl-Pak^®^ bag were used to confirm moisture content, electrical conductivity, and pH analysis under laboratory conditions. These samples were kept cold at 4°C until processed.

### Microbial Community Analysis

Total DNA was extracted from approximately 1 g of soil using a modified version of the CTAB (cetyl-trimethyl-ammonium-bromide) bead-beating method (Coyne et al., 2001). For each batch of DNA extractions, a negative control was included to ensure the identification of possible contamination. DNA concentration was determined using the Qubit dsDNA HS Assay Kit (Thermo Fisher Scientific, United States). The bacterial and archaeal microbial community was targeted through the V4 region of the 16S rRNA gene was amplified in triplicate using the fusion-primer set 515F/806R (Parada et al., 2016) and sequenced using Ion PGM chemistry. Briefly, 20 μL PCR reactions each contained: 1 ng of total DNA, dNTPs (240 μM), MgCl_2_ (6 mM), bovine serum albumin (0.24 μM), forward and reverse fusion primers (0.2 μM), 1 U of Platinum Taq polymerase (Invitrogen Inc., Carlsbad, California) and PCR buffer (1.2×). The following PCR conditions were used: 94°C for 3 min, followed by 30 cycles at 94°C for 45 s, 50°C for 1 min, 72°C for 1.5 min, and a final extension step at 72°C for 10 min. For each PCR run, negative amplification was confirmed for each DNA extraction batch control. Triplicate PCR amplicons were pooled, and the expected amplicon size was confirmed via electrophoresis with a 1% agarose TAE gel. PCR products were cleaned and each sample concentration was normalized with SequalPrep™ (Thermo Fisher Scientific, United States). Normalized samples were pooled at an equimolar concentration into a single library for sequencing. Amplicon sequencing was performed using the Ion PGM™ System for Next Generation Sequencing (Thermo Fisher Scientific, United States) at the Waikato DNA Sequencing Facility (University of Waikato, New Zealand).

Raw sequences were filtered with Ion PGM™ software to remove low-quality and polyclonal reads. The remaining sequences were processed using a combination of Mothur (v.1.40.5) and USEARCH 10 (v10.0.24) software (Schloss et al., 2009; Edgar, 2010, 2013). Forward and reverse primers were identified within the sequences and trimmed using the python script fastq_strip_barcode_relabel.py supplied by UPARSE (v10.0.240). Sequences without forward and reverse primers were discarded. The remaining sequences were trimmed based on the length and the number of homopolymers sourced by Mothur script (Schloss et al., 2009). All reads with expected error rates higher than 2.5 were discarded using USEARCH (Edgar, 2010), and all reads were truncated to 350 bp. Through dereplication, unique sequences were identified and their abundances quantified. Sequences were sorted, singletons removed, and the remaining sequences were clustered into representative OTUs using the UPARSE-OTU algorithm combined with the GOLD database to detect and remove chimeras (Bernal et al., 2001; Edgar, 2013). Reads were finally clustered into operational taxonomic units (OTUs) using UCLUST with a similarity threshold of 97%. Sequences that did not map to any OTU were discarded. Taxonomy was inferred using SINA (v1.2.11) and the SILVA SSU database (v 138) (Pruesse et al., 2012). A 0.005% cutoff was applied to the raw OTU counts across the dataset to remove poorly represented OTUs (Bokulich et al., 2013). Filtered OTUs were subsequently removed from the sequence Fasta file. Sequences were aligned using a Multiple Alignment using Fast Fourier Transform with default settings (MAFFT v7.429-gimkl-2020a) and a phylogenetic tree was generated using FastTree (v2.1.11).

### Statistical Analysis

All statistical and visualization analyses were computed in R (v3.5.2, R Core Team, 2000) using the following packages: phyloseq (v1.26.1) (McMurdie and Holmes, 2013), vegan (v2.5) (Oksanen et al., 2012), picante (v1.8) (Kembel et al., 2010), ggplot2 (v3.2.1) (Wickham, 2016), ggpubr (v0.4.0), compositions (van den Boogaart and Tolosana-Delgado, 2008), and randomForest (v4.6) (Liaw and Wiener, 2002). Alpha diversity was calculated on a non-rarefied dataset using richness and phylogenetic diversity (PD) indexes. Differences in library sizes were tested on both rarefied and non-rarefied data with no significant impact on the data interpretation. Diversity differences between transect zones and lakes were tested using one-way ANOVA. Normality assumptions were tested using a Shapiro-Wilk test, and the assumption of homogeneity of variances was tested using a Levene’s test. For the relationship between the microbial diversity and the log transformed moisture content and electrical conductivity, correlation analyses (Pearson) were applied. For beta diversity analysis we transformed the data using a total sum normalization. Beta diversity was calculated using weighted UniFrac phylogenetic pairwise distances (Lozupone and Knight, 2015) and visualized in a principal coordinates analysis (PCoA). A PERMANOVA analysis, using Adonis function in vegan package (Oksanen et al., 2012), tested the dissimilarities among communities from different groups (lakes, and zones within the wetness gradients). The variation between samples from the three wetness zones described along the transects was tested using a permutational multivariate analyses of dispersion (betadisp) from vegan package (Anderson et al., 2006). A Random Forest model (Liaw and Wiener, 2002) was used to detect the most reliable and relevant top 20 OTUs to predict the different zones along the wetness gradients. Raw counts were transformed using a centered log-ratio (clr) before classification and regression models. OTUs were selected based on the MeanDecreaseAccuracy values, which measures the extent to which a variable (OTU) improves the accuracy of the forest in predicting the classification. Higher values indicate that the OTU improves the prediction. To evaluate the phylogenetic community assembly among species within each sample we calculated the mean nearest taxon distance (MNTD) and the nearest taxon index (NTI) using “taxa.labels” null model, with 999 iterations, using the function “ses.mntd” from the R package picante. The NTI was quantified as the number of standard deviations that the observed MNTD was from the mean of the MNTD null distribution, multiplying by –1. For a single community, observed NTI values > + 2 or < −2 indicate phylogenetic clustering of species or phylogenetic overdispersion, respectively. NTI values between −2 and 2 usually indicates the influence of a stochastic assembly (Stegen et al., 2012). To compare community assembly processes along the moisture gradients, we calculated the Beta Nearest Taxon Index (βNTI). Following Stegen et al. (2013), the βNTI is the number of standard deviations that the observed beta mean nearest taxon distance (βMNTD) is from the mean of the null distribution. It indicates how much the observed difference between a pair of communities differs from a null distribution. Similarly to NTI, values > + 2 or < −2 indicate phylogenetic clustering of species or phylogenetic overdispersion, respectively, while values between −2 and 2 usually indicates the influence of a stochastic assembly (Stegen et al., 2013). β-NTI pairwise comparisons were plotted against increasing differences in moisture content. A Euclidean distance matrix was calculated using pH, electrical conductivity, water activity, moisture content, and elevation log-transformed and normalized data. ANOSIM was performed on the resemblance matrix to test the significance of the dissimilarities between the predefined zones of the wetness gradients. Sampling locations were plotted using Quantarctica (v3.1) (Matsuoka et al., 2021).

## Results

### Characterization of Space-for-Time Wetness Transects

**FIGURE 2:**
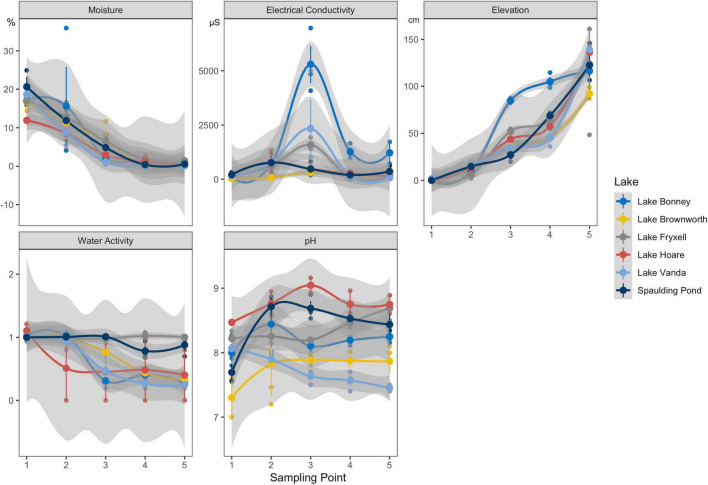
Soil moisture content (%), electrical conductivity (μS), elevation (cm), water activity (WA), and pH profiles along the space-for-time transects defined from all major lakes (*n* = 3, except for Lake Hoare *n* = 2) in Taylor and Wright valleys.

Seventeen geochemically defined space-for-time transects were parameterized using measurements of elevation, soil moisture content, electrical conductivity, pH, and water activity from both dynamic (*n* = 11 transects; Lake Vanda, Lake Bonney, Lake Hoare, and Lake Fryxell) and static (*n* = 6 transects; Lake Brownworth and Spaulding Pond) lakes across the Wright and Taylor Valleys (Figure 1, Table 1, and Supplementary Figure 2). Across all transects, elevation increased from wet (sampling point 1) to dry (sampling points 4 and 5) soils (from 0 to 135 ± 19 cm), and soil moisture content decreased in the same direction (from 17.25 ± 3.07 to 0.54 ± 0.54%) (Figure 2 and Table 1). Electrical conductivity was lowest in wet and dry soil samples (138 ± 81 μS and 341 ± 443 μS, respectively), achieving the highest values and variation within the transition zone (938 ± 1,591 μS) (Figure 2 and Table 1). Water activity decreased from wet to dry soils in Lake Vanda, Lake Bonney, Lake Brownworth, and Lake Hoare and remained stable across the wetness gradient in Spaulding Pond and Lake Fryxell (Figure 2 and Table 1). The variability in soil pH across the wetness gradients was lake-specific (Figure 2 and Table 1). The significance of the distinction between the three wetness zones (wet, transition, and dry) was confirmed with non-parametric testing (ANOSIM, *R* = 0.69, *p*-value < 0.01, 999 permutations) (Supplementary Figure 3).

### Sequencing Results and Quality Control

After filtering out low-quality and short sequence reads, we obtained a total of 2,084,605 sequence reads and 4,098 OTUs at 97% sequence similarity (Supplementary Table 1). We then filtered the low abundant OTUs using a 0.005% relative abundance cutoff across the entire OTU dataset and removed 72% of the initial OTUs. The remaining 28% (1,133 OTUs) comprised 97% of the initial reads. A significant correlation exists between the ordinations of the initial and filtered datasets (m12 squared: < 0.001; Procrustes Correlation: 0.99; *p*-value < 0.01, 999 permutations). We removed thirty-two OTUs classified as Chloroplast using the SILVA database, leaving a dataset containing 1,101 OTUs for downstream analysis. Sixteen percent of the remaining OTUs were unclassified at the phylum level. The Procrustes Correlation between the ordination matrices with and without the unclassified phyla was significant (m12 squared: < 0.001; Procrustes Correlation: 0.99; *p*-value < 0.01, 999 permutations). As a result, we retained the unclassified OTUs in the analysis.

### Microbial Community Diversity Along Space-for-Time Wetness Transects

**FIGURE 3:**
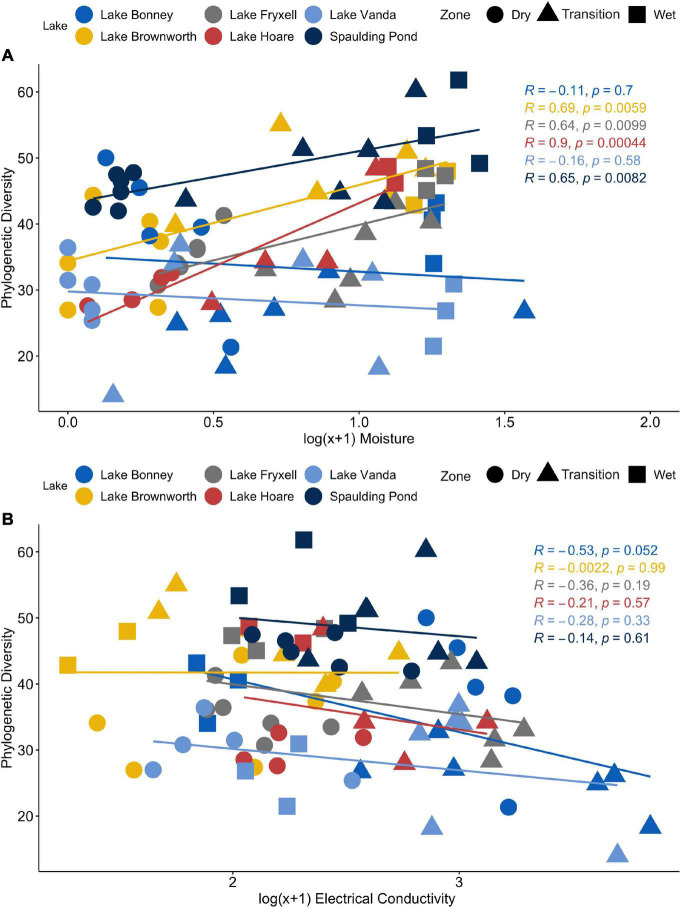
**(A)** Relationship between Phylogenetic Diversity (PD) and log transformed moisture content measurements across the SFT transects from Lake Brownworth, Lake Bonney, Lake Fryxell, Lake Hoare, Spaulding Pond, and Lake Vanda. **(B)** Relationship between Phylogenetic Diversity (PD) and log transformed electrical conductivity measurements across the SFT transects from Lake Brownworth, Lake Bonney, Lake Fryxell, Lake Hoare, Spaulding Pond, and Lake Vanda.

We observed significant differences in species phylogenetic diversity (PD) within the dry, transition, and wet transect zones from each lake (Supplementary Figure 4; *p*-value < 0.01). Spaulding Pond consistently presented the most diverse community along the wetness gradients and Lake Vanda the least diverse community (Supplementary Figure 4). Except for Lake Vanda and Lake Bonney, PD correlated positively with soil moisture content (*p*-value < 0.05; Figure 3A). Soil electrical conductivity correlated negatively with PD (*p*-value < 0.05; Figure 3B) only in Lake Bonney transects, likely driven by the comparatively high electrical conductivity (5,300 μS) measurements.

### Microbial Community Composition Along Space-for-Time Wetness Transects

The soil microbial community was represented by 24 phyla, with 98% of reads classified within the top 10 phyla (Supplementary Figure 5). These dominant 10 phyla maintained their representation across all lakes sampled, with a variation in relative abundance reflective of the moisture zone. *Bacteroidota* (0.31 ± 0.08), *Proteobacteria* (0.24 ± 0.08), and *Cyanobacteria* (0.19 ± 0.11) were consistently abundant in wet soils. *Bacteroidota* (0.28 ± 0.09), *Proteobacteria* (0.18 ± 0.10), and *Actinobacteriota* (0.15 ± 0.13) were highly abundant in the transition zones. Dry zones were dominated by members of *Actinobacteriota* (0.30 ± 0.07), *Bacteroidota* (0.20 ± 0.05), *Proteobacteria* (0.11 ± 0.03) and *Acidobacteriota* (0.10 ± 0.06) phyla. In Lake Bonney, taxa affiliated with the phyla *Cyanobacteria* and *Firmicutes* presented a relatively high abundance in transition zone (0.16 ± 0.08 and 0.14 ± 0.16, respectively) and dry samples (0.36 ± 0.09 and 0.09 ± 0.09, respectively).

### Microbial Community Structure Along Space-for-Time Wetness Transects

Community beta-diversity from communities from the dry zones of space-for-time transects varied significantly between lakes (PERMANOVA, *R*^2^ = 0.69, *F* = 11.55, *p*-value < 0.01; Supplementary Figure 6A), sharing only 32% of the OTUs (Supplementary Figure 7A). Microbial communities from the wet zones of space-for-time transects were significantly different between lakes (PERMANOVA, *R*^2^ = 0.73 *F* = 4.47, *p*-value < 0.01; Supplementary Figure 6B), sharing only 16% of the OTUs (Supplementary Figure 7B).

**FIGURE 4:**
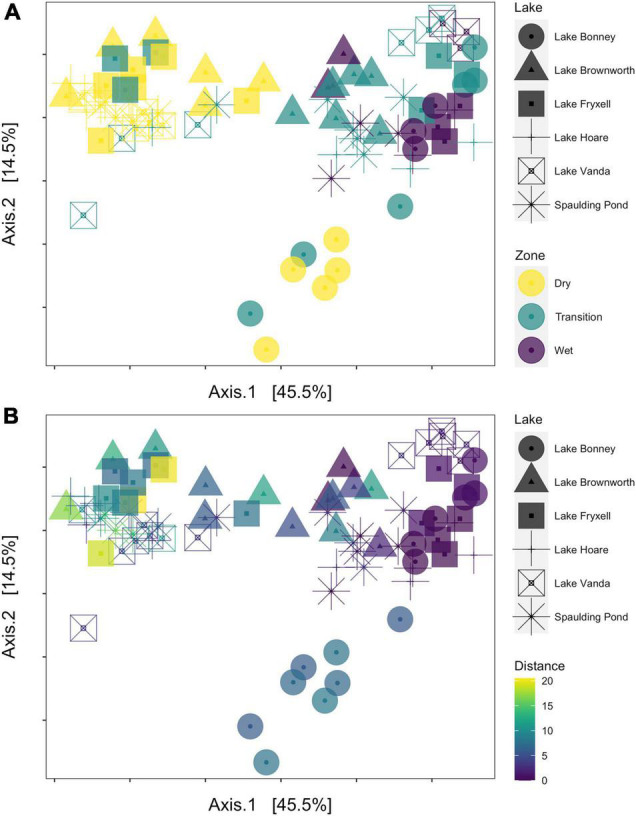
Principal coordinate analysis (PCoA) of microbial community compositional data based on a weighted UniFrac distance matrix. **(A)** The samples are colored by transect zone. **(B)** The samples are colored according to their distance from the lake shore.

**FIGURE 5:**
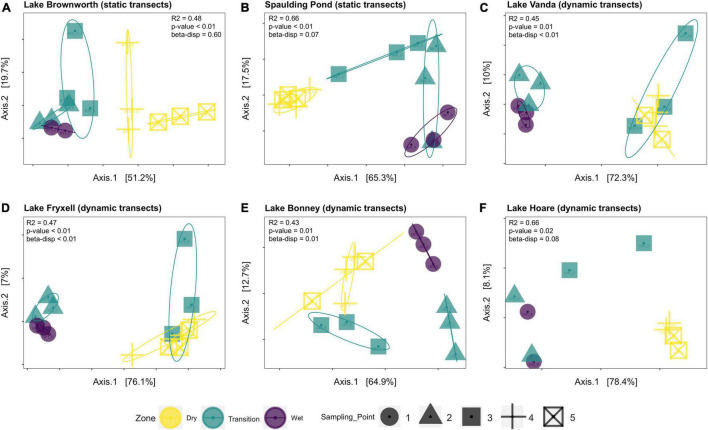
Principal coordinate analysis (PCoA) of microbial community data based on a weighted UniFrac distance matrix for each lake along each transect: **(A)** Lake Borwnworth; **(B)** Spaulding Pond; **(C)** Lake Vanda; **(D)** Lake Fryxell; **(E)** Lake Bonney; **(F)** Lake Hoare. The significance between weighted UniFrac distance was calculated using PERMANOVA. The assumption for homogeneity of group dispersion was tested with betadisper function. Ellipses on the PCoA plot highlight the deviations from the mean for each clustered group of samples representative of different zones of the transects (95% confidence level).

Along the space-for-time transects, communities collected in the dry zone were structurally distinct from those collected in the wet zone (PERMANOVA, *R*^2^ = 0.27, *F* = 14.11, *p*-value < 0.01) (Figure 4). For each lake, significant clustering of the communities by zone was identified (Figure 5). However, only the Lake Brownworth Spaulding Pond and Lake Hoare transects met the premise for homogenous dispersion (Figures 5A,B,F; beta-dispersion > 0.05). For all dynamic lake transects (apart from Lake Hoare), the dispersion of communities within the transition zone was significantly different (beta-dispersion < 0.05) (Supplementary Table 2). Except for Lake Hoare, the transition zone community across all dynamic lake transects was segregated into two clusters, with one of the clusters intercepting with the dry soil community cluster on sampling point 3 (ellipses at 95% confidence level) (Figures 5C–E). The latter was not observed in the stable lake transects collected from Lake Brownworth (Figure 5A) or Spaulding Pond (Figure 5B).

**FIGURE 6:**
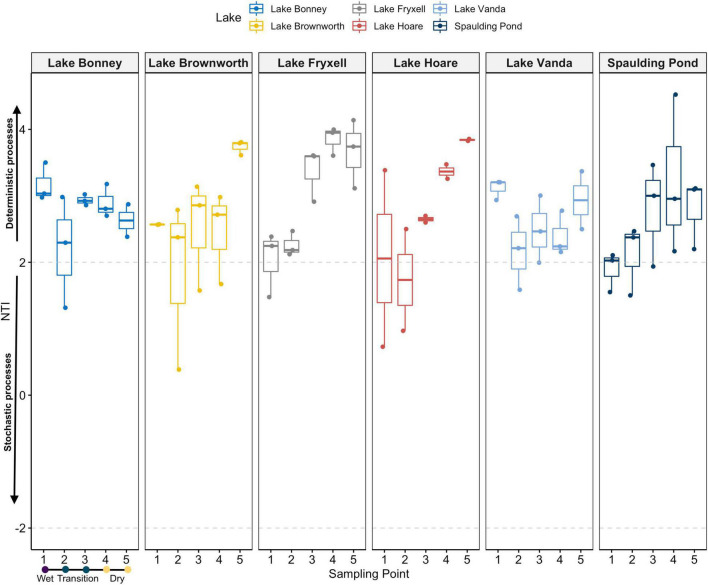
NTI values for each single community sampled along space-for-time transects at each lake. Dashed gray lines at the −2 and + 2 values delimitate the significance thresholds from the null expectation. NTI values < −2 or > + 2 suggests that phylogenetic turnover is less or greater than the null expectation, and it is related to deterministic processes. NTI values ranging between –2 and 2 is usually considered signifying the influence of stochastic assembly.

Phylogenetic community composition within each sample revealed a trend leaning toward a deterministic assembly of the community governed by environmental selection (NTI > 2) (Supplementary Figure 8A). That effect was higher, and consistent in the dry soils (sampling point 5) and tended to decrease toward the wet zone (sampling point 1) of the gradient, except for Lake Bonney and Lake Vanda, where no trend was identified (Figure 6). In contrast to NTI values, βNTI values distribution showed a median close to 0 but with skewed distribution stretching beyond the + 2 significant threshold (Supplementary Figure 8A). This reflects the influence of deterministic processes for a given pairwise comparison. However, pairwise comparisons regressed against differences in moisture content indicates a large influence of stochasticity with a weak relation to moisture content (Supplementary Figure 8B).

### Microbial Taxa as Sentinels for Change

**FIGURE 7:**
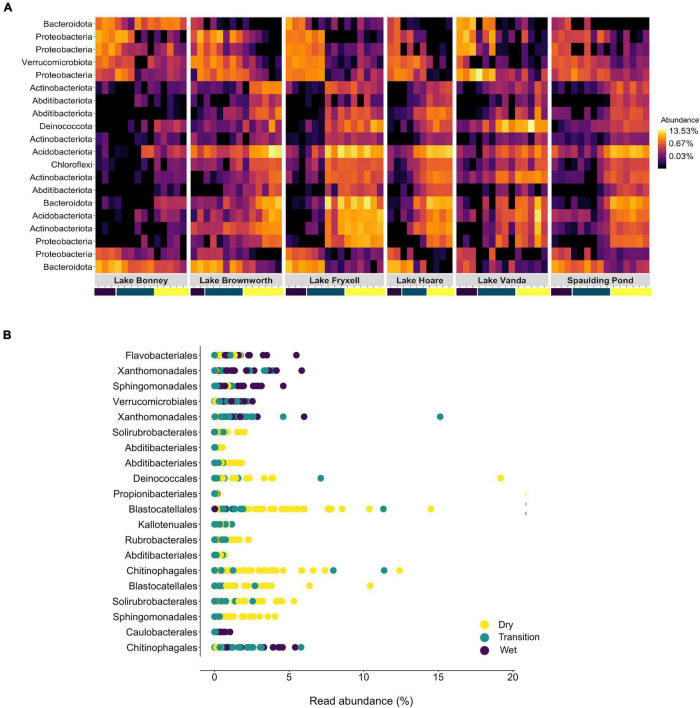
**(A)** Heatmap of the top 20 bacterial OTUs classified by Random Forest classification analysis as the most important to discriminate between the different zones of lake transects. Each row represents each OTU at the phylum level, and the colour of the box indicates the relative abundance of each OTU, with yellow depicting high relative abundance and black low relative abundance. **(B)** Rank abundance plot of the correspondent top 20 OTUs identified across the different zones of the SFT transects at the order level.

Soil moisture content was the primary non-categorical variable driving microbial community structure patterns (PERMANOVA, *R*^2^ = 0.25, *F* = 26.48, *p*-value < 0.01; Table 2). A random forest approach identified that 64.8% of the variance in the community could be explained by moisture content (RF, no of trees: 10001, mean of squared residuals 19.88). Taxa affiliated to *Actinobacteriota, Chloroflexi, Deinococcota, Verrucomicrobiota, Proteobacteria, Acidobacteriota, Abditibacteriota*, and *Bacteroidota* were the major taxa driving differences between wet and dry soils (out-of-bag estimate of error rate for the transect section classification: 24.4%) (Figure 7A). OTUs affiliated to *Actinobacteria*, order *Solirubrobacterales*; *Chloroflexi*, order *Kallotenuales*; *Deinococcota*, order *Deinococcales; Acidobacteria*, order Blastocaellales, and *Abditibacteriota*, order *Abditibacteriales*, were exclusively associated to dry and transition zone soils (Figure 7B and Supplementary Table 3). Our model also identified the OTU assigned to *Verrucomicrobiota*, order *Verrucomicrobiales* (genus *Luteolibacter*) as an important OTU to classify wet soils (Figure 7B and Supplementary Table 3). Members of *Bacteroidota* and *Proteobacteria* phyla were associated with both dry or wet soil conditions. *Bacteroidota* taxa related to wet soil conditions belonged to the *Flavobacterium* and *Ferruginobacter* genus (order *Flavobacteriales* and *Chitinophagales*), whereas the genus *Segitobacter* (order *Chitinophagales*) was more abundant in dry soils. Within *Proteobacteria*, 4 OTUs affiliated to the genus *Sphingorhabdus* (order *Sphingomonadales*), *Thermomonas and Pseudoxanthomonas* (order *Xanthomonadales*), and genus *Brevundimonas* (order *Caulobacterales*) were associated with wet soil conditions, whereas the genus *Sphingomonas* (order *Sphingomonadales*) were associated with the dry zone of the gradient (Figure 7B and Supplementary Table 3).

**TABLE 2:** PERMANOVA test using soil moisture content, pH, and electrical conductivity on microbial communities along the wetness transects.

	F model	Statistic R2	Significance (*p*-value)
Moisture (%)	26.48	0.25	<0.01
pH	1.36	0.02	0.22
Electrical conductivity (μS)	5.28	0.06	<0.01

## Discussion

Here we sought to validate the application of space-for-time as a reliable approach to forecast the impacts of climate-related hydrological changes on the terrestrial microbiome in a polar desert. We chose the soil ecosystem of the MDV as a natural, trophically simple, and well-characterized system where single environmental drivers (such as water availability) have a profound and quantifiable impact on the resident microbial community (Van Horn et al., 2014; Niederberger et al., 2015, 2019; Buelow et al., 2016; Lee et al., 2018; Bottos et al., 2020; Coyne et al., 2020; Ramoneda Massague et al., 2021). Our sampling approach starts by highlighting the importance of selection of sites along spatial gradients based on field determinations of critical environmental drivers of species distributions and their patterns across space. This will help to select sites at consistent points along different ecotones, which can be missed when arbitrary measures such as distance are chosen (Figure 2). Secondly, we point out the importance of repeating space-for-time transects across multiple units within the ecosystem to fully harness the characteristics of environmental variability. Lastly, we considered the detailed historical background on ecosystem response to climate change and defined transects in relatively stable and dynamic environmental scenarios. The MDV offers access to multiple lake systems with variable rates and timing of change. Closed-basin lakes, in particular, are quite sensitive to climate change, which is reflected by historic and current changes in water level and salinity (Castendyk et al., 2016; Levy et al., 2018). Across all sampled lakes Lake Vanda and Lake Bonney have experienced the biggest water level rise, followed by Lake Fryxell and Lake Hoare (Levy et al., 2018). Open-basin lakes, in contrast, may change at slower rates due to an established outlet that rapidly drains glacial meltwater and groundwater flow (Levy et al., 2018). Transects defined in open-basin lakes set the background state for longer periods of stability, whereas transects defined in closed-basin lakes mirror more recent and predicted hydrological disturbances in the system.

### Historical Legacy of Water Availability Reflected by the Structure and Composition of Microbial Communities

By comparing geochemically defined space-for-time transects from lake systems with relatively stable or dynamic water levels, we were able to resolve the legacy impacts of water presence on microbial community structure (Figure 4). Only within transects defined from Lake Brownworth, and to a lesser extent Spaulding Pond (static transects), did the microbial community display specific structural patterns reflecting the three geochemical zones identified (Figures 5A,B). The high degree of zone specificity (wet, transition, and dry) in community structuring from these lakes aligns with an extended period of more stability due to the presence of an outflow (Levy et al., 2018). In contrast, from the dynamic closed-basin lake transects, the clear spatial segregation of the transition zone communities into one of “dry” or “wet”-like community could reflect a response related to legacy of water level changes in these lakes, not detected by the defined geochemical metrics, which respond on a different time scale. Particularly in Lake Vanda, Lake Bonney, and Lake Fryxell (Figure 5C–E), which have risen between 1.6 and 4.1 m from 2001 to 2017 (Levy et al., 2018), no distinct transitional zone community was recognizable (Figure 7A). Our findings support recent monitoring of Lake Vanda water levels, which indicate that this lake has been continually rising at a rate of 22 cm/year since 1947 (Castendyk et al., 2016). The lack of zone specificity in the microbial community structure at this lake likely reflects that at the time of sampling, microbial communities collected at the geochemically defined transition between transition and dry zones were likely to have been recently wet (potentially hours to days) and remain similar to a dry soil community. In contrast, communities exposed to wetness for more extended periods resemble a more long-term wet soil community. Similar temporal shifts were observed by Ramoneda Massague et al. (2021) in moat samples collected along a depth gradient in Lake Vanda. Microbial assemblages collected from the higher depths diverged from those collected at the lake shore, being likely influenced by pre-existing terrestrial microbes in recently inundated soils (Ramoneda Massague et al., 2021). The consistency of our results across closed-basin lake transects suggests that a geochemically defined space-for-time sampling approach can be applied across polar systems to monitor change through the lasting legacy impacts of water availability on assembly mechanisms that shape extant microbial community structure.

Upon observing different spatial structuring of microbial communities, we evaluated whether microbial community assembly patterns within and between communities along the moisture gradient were driven by deterministic or stochastic processes, or by a combination of both. Contrary to a previous observation (Lee et al., 2018), the NTI values observed for each sample across all 17 geochemically defined space-for-time transects indicate that the degree of phylogenetic relatedness among the individuals in a community is likely driven by environmental selection (NTI > 2) (Figure 6). In the MDV, the dry soils are typically oligotrophic, with minimal water content (<2%) and high salt concentrations (Cary et al., 2010), which collectively exclude a significant range of taxa that cannot tolerate such selective pressures (Chase, 2007; Stegen et al., 2012). Nonetheless, overall patterns of community assembly along increasing differences in moisture content (βNTI values) suggest a considerable but variable influence of stochastic and, to a lesser extent, deterministic processes, with a weak relation to differences in moisture content (Supplementary Figure 8). While this was surprising considering the NTI values, it suggests that, at a regional scale, community assembly is driven by stochastic processes, but phylogenetic differences within communities are driven by deterministic processes that favor the occurrence of closely related taxa. Such a pattern could indicate that microbial traits that confer adaptability are phylogenetically not well-conserved across the region. Alternatively, it could also result from the distribution of spore-forming, or dormant bacteria in dry or highly conductive soils (Lee et al., 2018), or from differences in diversity between lakes or along the gradients. Overall, we suggest that deterministic and stochastic processes play a role in community assembly, however, further extensive analyses are required to understand how deterministic and stochastic components change communities as moisture content changes in the soils.

The impact of moisture as a key driver of microbial diversity was evident at all lakes studied except in Lake Bonney and Lake Vanda (Figure 3A). The presence of water in an available state for life has quantifiable impacts on soil microbial diversity within polar deserts (Niederberger et al., 2015, 2019; Buelow et al., 2016; Bottos et al., 2020). Nonetheless, its positive impact can be suppressed by the effect of soil conductivity (Van Horn et al., 2014; Feeser et al., 2018; Bottos et al., 2020; George et al., 2021). This was the case of Lake Bonney, where the high levels of soil conductivity affected the microbial diversity along the wetness gradient (Figure 3B), creating a niche that only allows for taxa capable of tolerating high osmotic stress (e.g., *Firmicutes*) to thrive. Lake level rise caused by increased glacial runoff in Lake Bonney facilitates the leaching and movement of accumulated meteoric salts, altering soil geochemistry, particularly pH and conductivity (Barrett et al., 2009). High conductivity measurements in these soils support our evidence for the greater prevalence of deterministic processes driving *within* community assembly in Lake Bonney and, to a lesser extent, in Lake Vanda, where the moisture content and relatively higher salt concentration in the soils may have a constant selective effect on the community. Both lakes are located further inland, where soils are characteristically older, more developed, and with higher salt concentrations and with different chemistry, compared to coastal soils (Bockheim, 2002). These could also be factors for such disparities in community diversity and composition along transects sampled from these two inland lakes. Although pH has been suggested to be a critical factor driving spatial patterns of microbial communities (Tripathi et al., 2018), in this study, pH changes within and between space-for-time transects did not have a significant effect on community diversity.

Specific phyla and orders of microbial taxa associated with long-term dry and wet conditions can provide a metric to assess early signs of change (Figure 7). Despite the variability in the structure of microbial communities among wet soil samples, OTUs affiliated with *Verrucomicrobiota* were consistent indicators of long-term wet conditions, whereas OTUs affiliated with *Proteobacteria* and *Bacteroidetes* were associated with both wet and dry conditions. On the other hand, *Actinobacteria*, *Acidobacteria*, *Chloroflexi*, and *Deinococcota* taxa significantly and consistently enriched communities from dry soils, corroborating our previous observations conducted in the MDVs (Niederberger et al., 2015, 2019; Zhang et al., 2020). A recent study by Lee et al. (2018) found similar taxonomic trends, with *Acidobacteria* and *Actinobacteria* being less abundant with higher moisture content. We then provide strong evidence that the presence of discrete taxa within the phyla *Actinobacteria* (family *Solirubrobacteraceae*), *Acidobacteria* (subdivision 4 genera *Blastocatella*), and *Deinococcota* (genus *Truepera*) are ubiquitously associated with long-term dry edaphic conditions. This could be a result of metabolic specialization in the shape of very limiting long-term dry, oligotrophic, and ionizing (UV radiation) conditions to life. Taxa belonging to the family *Solirubrobacteraceae* are currently known for their capability to metabolize atmospheric trace gases contributing to primary production in the dry oligotrophic soils (Ji et al., 2017), and members of the genus *Truepera* are known for being extremely resistant to ionizing radiation, which is considered a strong stress factor in arid desert soils (Cary et al., 2010). The alleviation of specific stress conditions will impose selective pressures on these highly adapted taxa. As such, any quantifiable taxonomic changes among these taxa can be developed as sentinels for increased temporal exposure to moisture within the MDVs soils, which from a structural standpoint shows a heightened sensitivity of the terrestrial Antarctic microbiome to hydrological changes.

### Validation of the Space-for-Time Approach

The space-for-time approach employs ecological structures within contemporary environmental gradients to project ecological responses to changes in the environment over time. Primarily applied to study slow ecological processes, this approach is informative when factors that drive community change are equivalent across space and time (Blois et al., 2013). From a microbial ecology perspective, the geochemical profile of a system is often highlighted as an important, if not the primary deterministic force driving community assembly, particularly in ecosystems with low trophic complexity or where diversity is environmentally constrained (Chase, 2007). Yet, sampling designs along spatial gradients are often conducted following distance points (Yang et al., 2014; Niederberger et al., 2015; Yan et al., 2017; Feeser et al., 2018; Lee et al., 2018) rather than being based on the spatial variability of geochemical factors, to which biological communities respond to, along a constrained gradient. The patterns of geochemical drivers across space may not be continuous along a gradient, therefore without characterizing them, geochemical boundaries that may drive community assembly within the gradient are likely to be missed, contributing to extant microbial community heterogeneity.

In an environmentally constrained system, such as the Antarctic polar deserts, we demonstrate that contemporary and historical environmental changes (e.g., water availability) can be resolved by subtle structural and compositional re-arrangements of the microbial communities along geochemically defined environmental gradients. These observations could only be depicted when sampling approaches are determined by the spatial variability of geochemical drivers along environmental gradients and replicated across comparable environments that differ in historical exposure to the driving factor. The validation of our space-for-time transects relies on the observed interdependence between structural and compositional community shifts across space, with the temporal exposure to wet conditions.

Given the accelerated rate of climate change across the globe (IPCC, 2021), the ongoing increasing lake levels (Castendyk et al., 2016; Levy et al., 2018), and the expected increase in snow and ground ice melt (Linhardt et al., 2019) will lead to drastic changes in the MDVs landscape, hydrology and ecology (Fountain et al., 2014). For instance, a recent study conducted in a high Arctic lake demonstrated that the taxonomic and functional diversity of dominant microbes can be impacted by an increase in glacial runoff as a consequence of rapid climate change (Colby et al., 2020). Our study, demonstrates that predictions of a wetter system will directly affect the stability of microbial communities currently adapted to dry and ultra-oligotrophic conditions, resulting in significant compositional and diversity changes across the system. Such environmental and taxonomical changes have the potential to lead to alterations in primary productivity, carbon and nitrogen cycling, and energy fluxes across the system (Coyne et al., 2020; Monteiro et al., 2020; Karen et al., 2021). Whilst microorganisms underpin the stability and functionality of polar deserts ecosystems, these are still rarely considered in policy development and lack any protective status within the Antarctic Treaty (Hughes et al., 2015). This study highlights that microbial communities are highly sensitive to change and closely reflect changes in the environment. Therefore, as in more temperate environments (Cavicchioli et al., 2019), microbial communities should be considered by policy-makers as primary sentinels for current and historical changes in the Antarctic systems.

## Data Availability Statement

The raw DNA sequences presented in this study can be found in the online repository: https://www.ncbi.nlm.nih.gov/bioproject/ under the BioProject accession number PRJNA764757.

## Author Contributions

SC, CL, and IH conceived and designed the project. SC and IH support for the project obtained. MM and IM performed by the all fieldwork analyses and sample collection. MM and AM conducted all subsequent laboratory work, DNA sequencing, and analyses. The manuscript was written by MM, AM, and SC with contribution and editing by CL, IM, and IH. All authors contributed to the article and approved the submitted version.

## Conflict of Interest

The authors declare that the research was conducted in the absence of any commercial or financial relationships that could be construed as a potential conflict of interest.

## Publisher’s Note

All claims expressed in this article are solely those of the authors and do not necessarily represent those of their affiliated organizations, or those of the publisher, the editors and the reviewers. Any product that may be evaluated in this article, or claim that may be made by its manufacturer, is not guaranteed or endorsed by the publisher.
