# Supplementary material for: Geochemically Defined Space-for-Time Transects Successfully Capture Microbial Dynamics Along Lacustrine Chronosequences in a Polar Desert
Source: Front Microbiol. 2022 Jan 31;12:783767. doi: 10.3389/fmicb.2021.783767 (PMC8841834; doi:10.3389/fmicb.2021.783767)
Supplement: Supplementary file 1 [file Data_Sheet_1.pdf]

Supplementary Material

Supplementary Figures

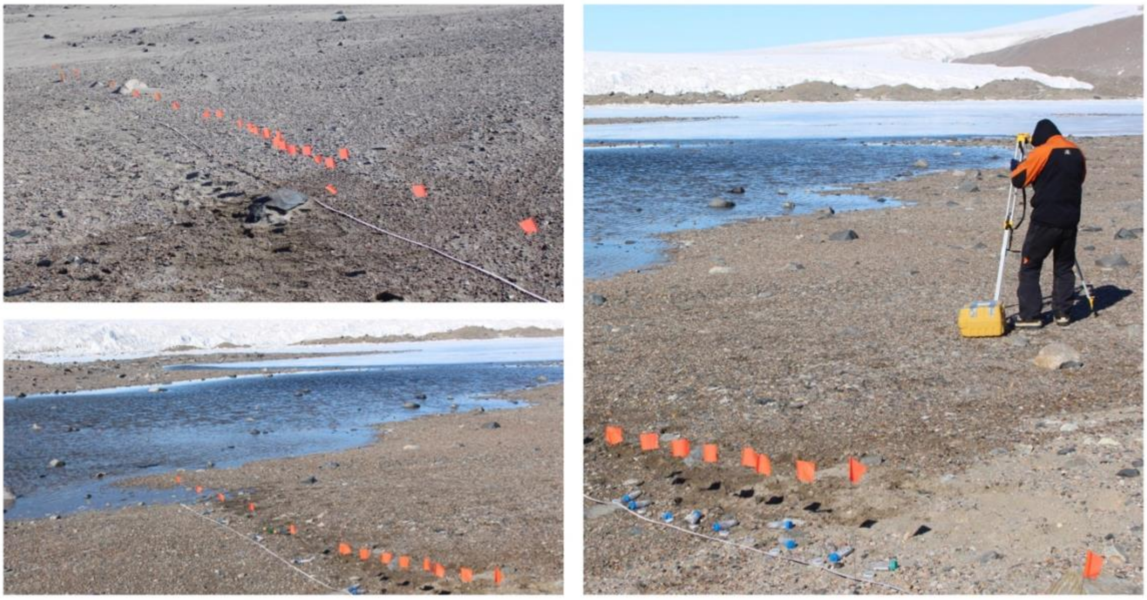

Figure S1 – Photos of space-for-time transects defined in Lake Brownworth during 2016/2017 field season. Before determining the sampling points, geochemical parameters of water activity, conductivity, and pH were measured along several points across the wetness gradients (averaging every 50 cm from the shore of the lake into the dry soil).

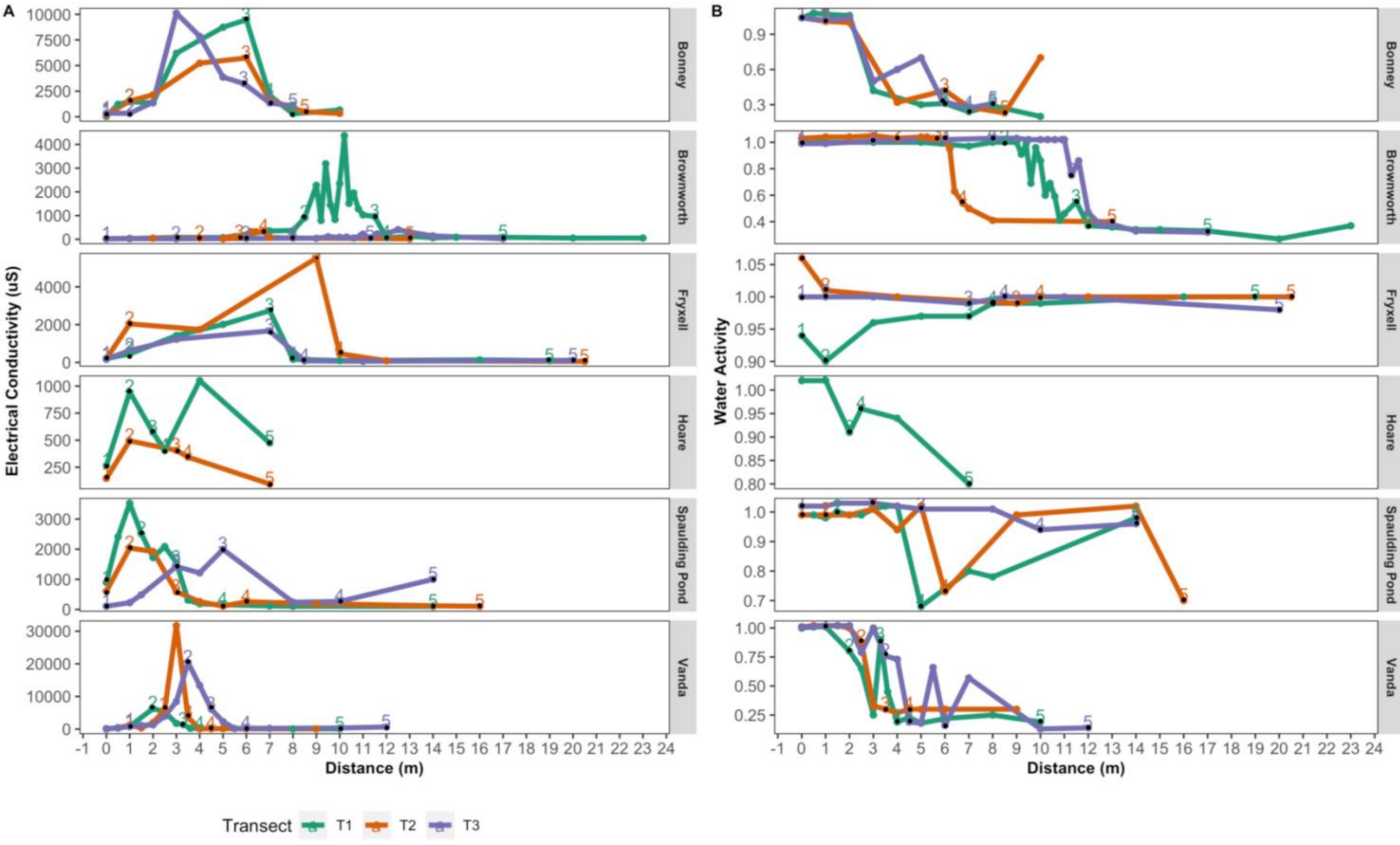

Figure S2 – Electrical conductivity (A) and water activity (B) profiles measured along the wetness gradient before defining the sampling points in Lake Bonney, Lake Brownworth, Lake Fryxell, Lake Hoare, Spaulding Pond and Lake Vanda. The location of each sampling point across distance can be depicted in the figures by the numbers.

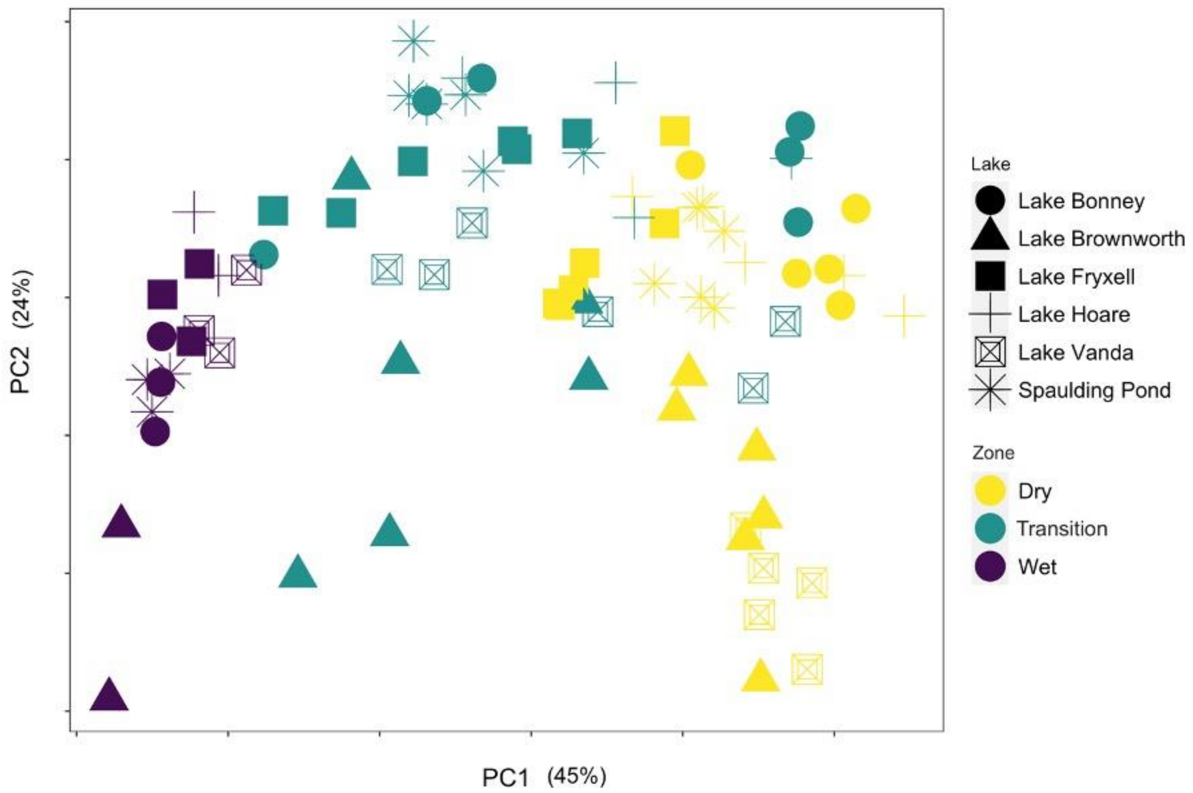

Figure S3 – PCoA ordinations based on the samples geochemical profiles. An Euclidean distance matrix was calculated using geochemical data collected in the field (elevation, pH, conductivity, water activity, and soil moisture content) after being log (x+1) transformed and normalized.

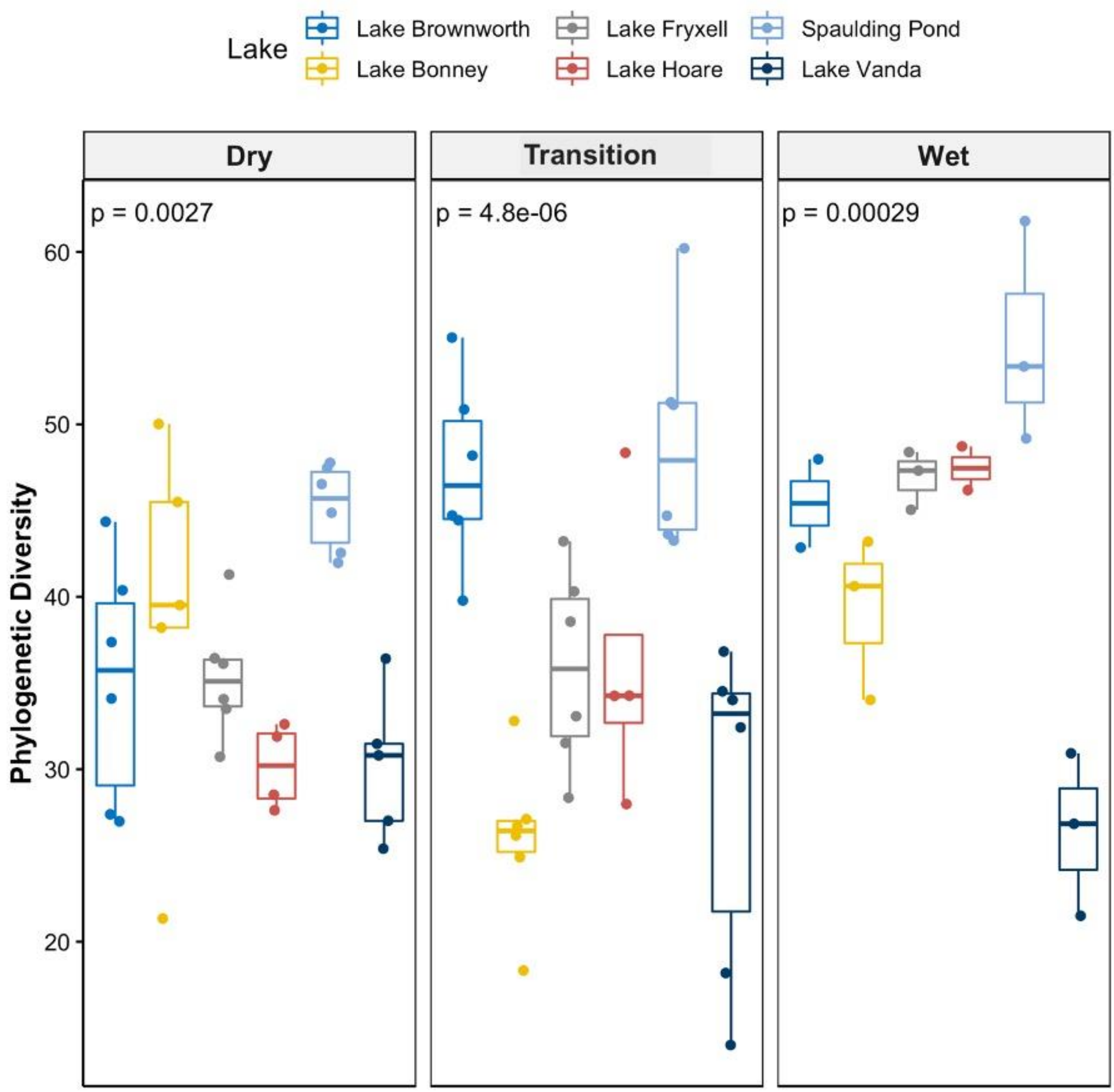

Figure S4 - Estimated OTU phylogenetic diversity (PD) of the microbial communities between different lakes on wet, transition, and dry sections of space-for-time transects. The error bars represent standard deviations of means.

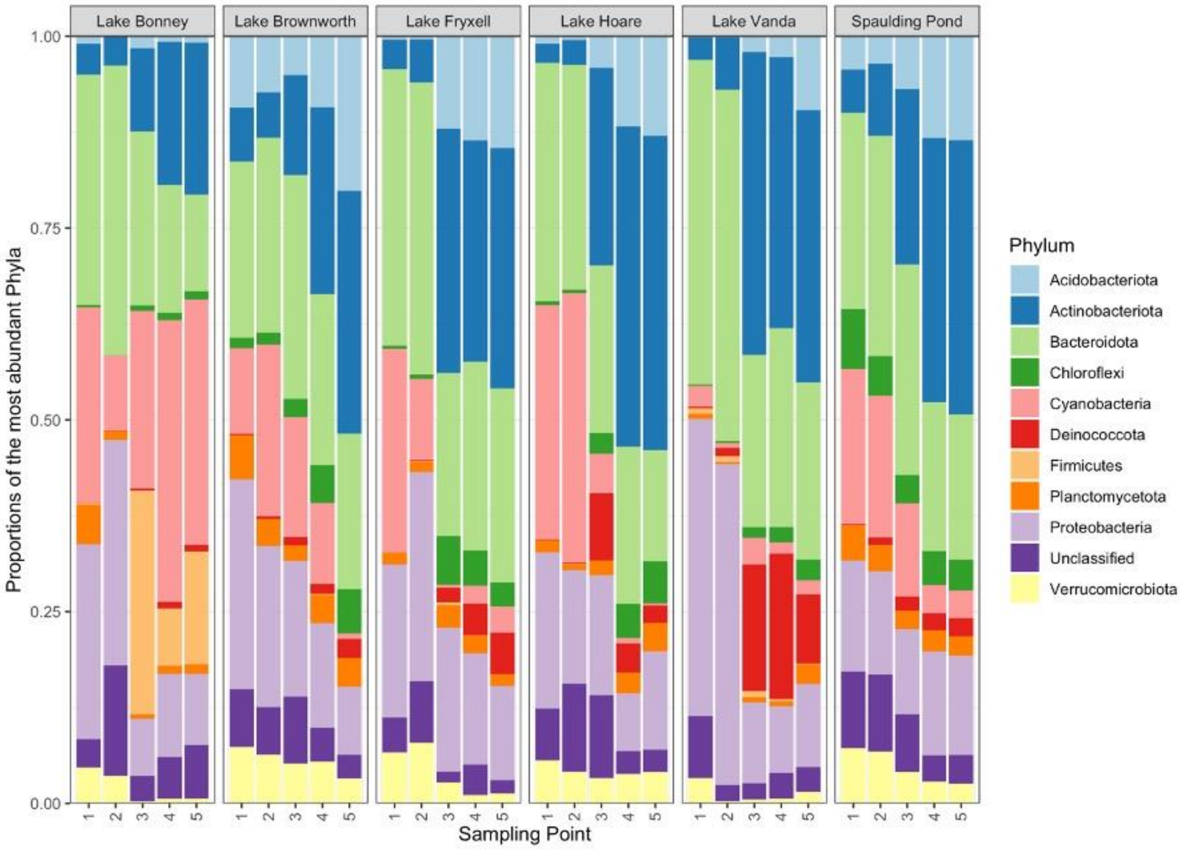

Figure S5 - Relative abundance of the most dominant bacterial phyla along space-for-time transects from each lake.

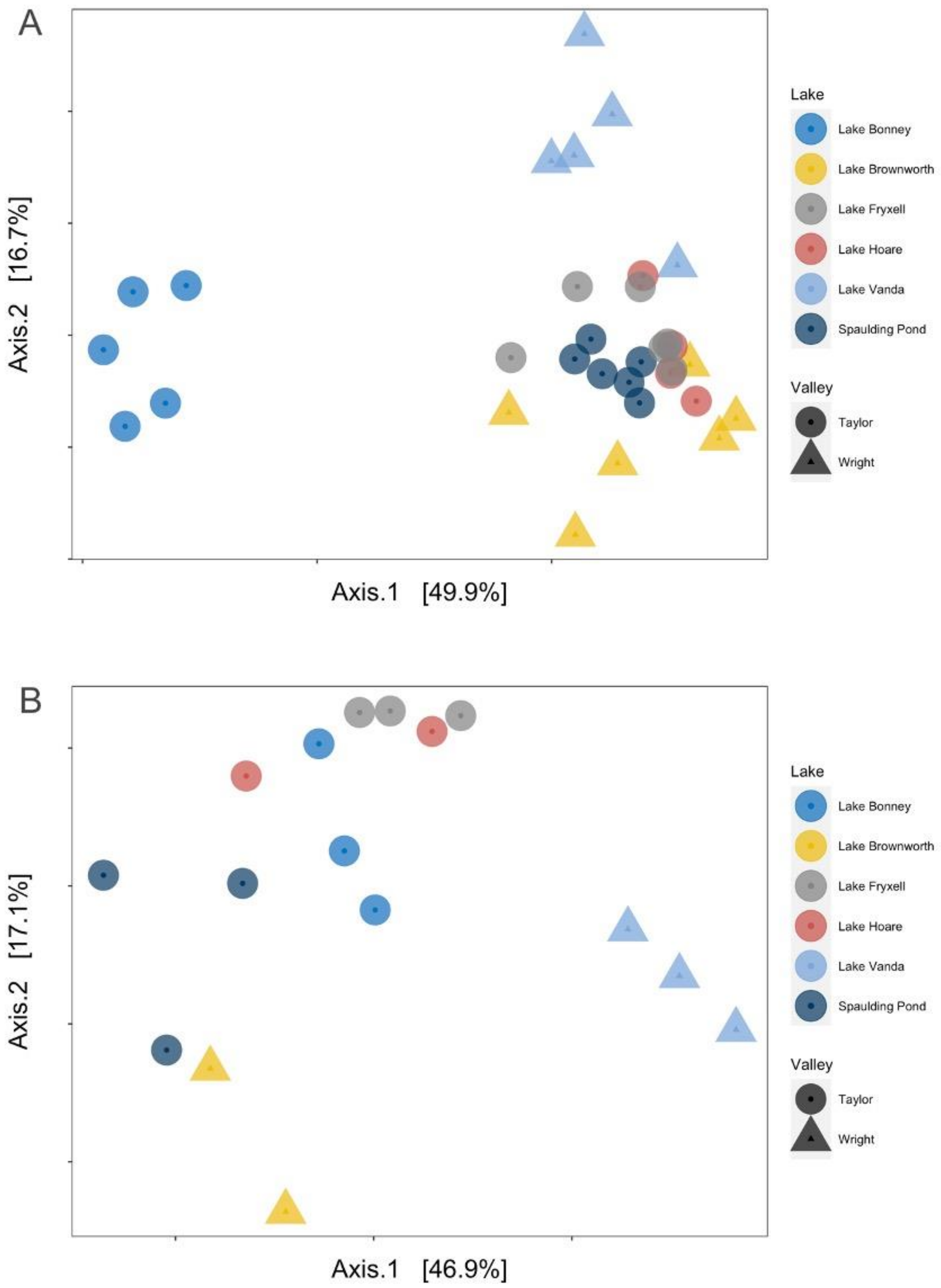

Figure S6 - Principal coordinate analysis (PCoA) of the microbial community compositional data from dry (A) and wet zones of all lake transects (B), using a weighted UniFrac distance matrix.

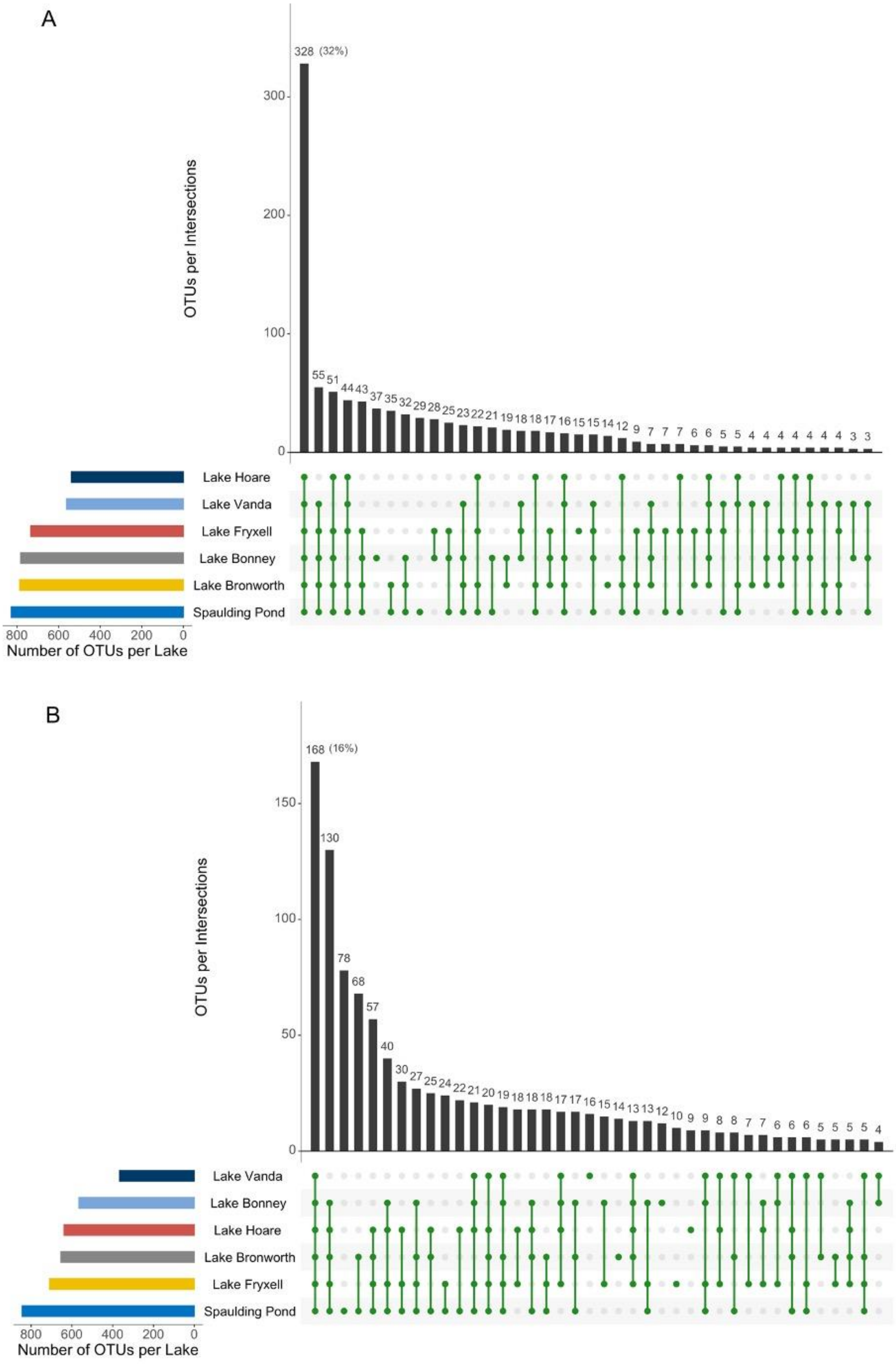

Figure S7 –UpSet plots showing the number of OTUs shared in the dry (A) and wet zones (B) of all lake transects.

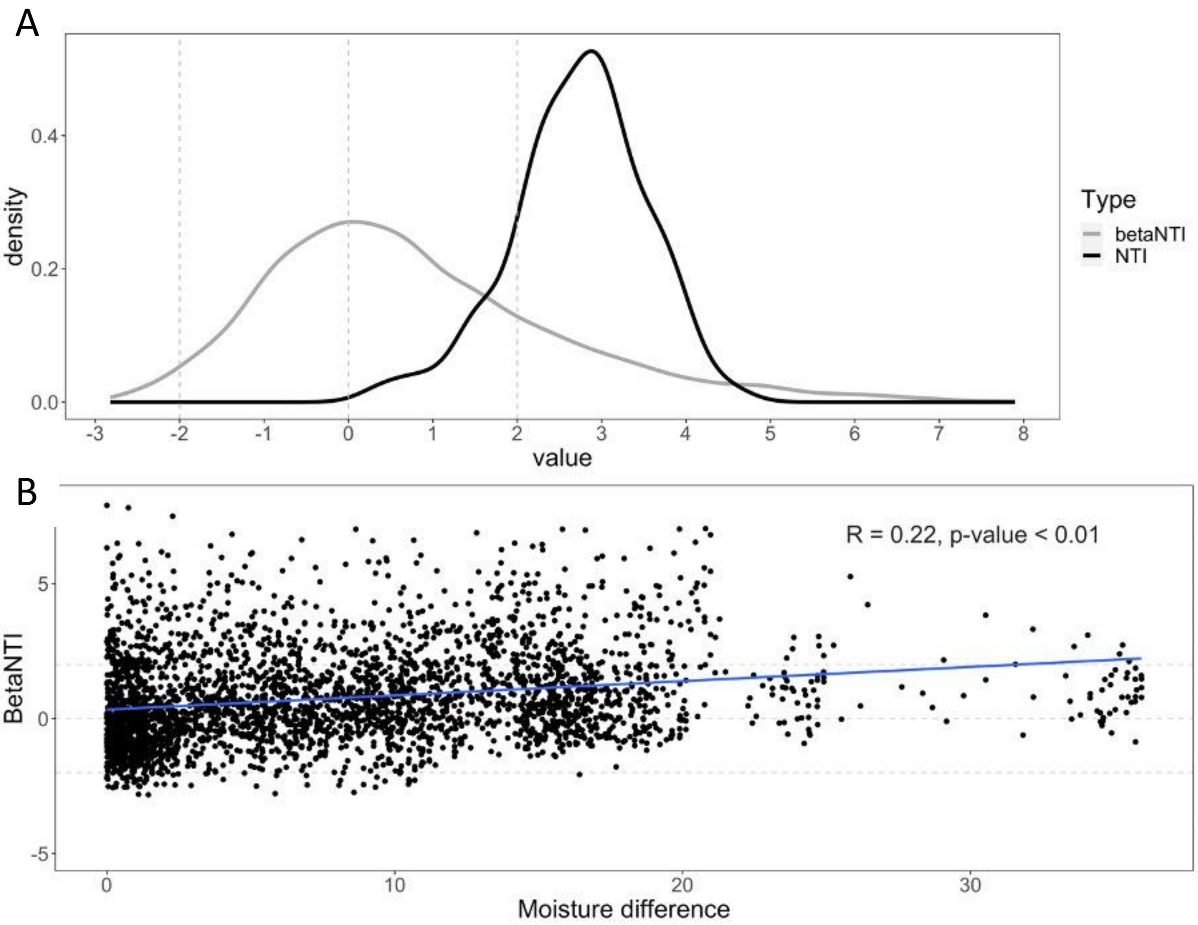

Figure S8 – A - density plot estimating the distributions of the nearest taxon index (NTI) and its between-community analogue (βNTI). Dashed grey lines at the −2 and +2 values delimitate the significance thresholds from the null expectation; **B** – βNTI values for all pairwise community comparisons regressed against the difference in moisture content between communities. The linear regression model is shown as the blue line. Dashed grey lines at the −2 and +2 values delimitate the significance thresholds from the null expectation.

Supplementary Tables

Table S1- Raw reads, sequencing and quality filtering statistics of 16S rRNA gene reads

ID	DNA yield (ng/g wet soil)	Raw reads	after QC	%	Length	after 0.005% culling	%	Transect	Sampling Point	Transect Section	Lake	Valley
BT3_0	421	71260	14670	21	350	11812	80.52	BT3	1	Wet	Lake Brownworth	Wright Valley
BT3_13	226	90447	23881	26	350	23704	99.26	BT3	5	Dry	Lake Brownworth	Wright Valley
BT3_4	250	97889	31299	32	350	28616	91.43	BT3	2	Transition	Lake Brownworth	Wright Valley
BT3_5.75	119	54774	13697	25	350	12706	92.76	BT3	3	Transition	Lake Brownworth	Wright Valley
BT3_6.75	890	67625	16549	24	350	15725	95.02	BT3	4	Dry	Lake Brownworth	Wright Valley
BT4_0	338	754	*all filtered out*	N/A	N/A	N/A	N/A	BT4	1	Wet	Lake Brownworth	Wright Valley
BT4_11.3	283	106305	36986	35	350	36375	98.35	BT4	3	Transition	Lake Brownworth	Wright Valley
BT4_12	55	64777	20699	32	350	20201	97.59	BT4	4	Dry	Lake Brownworth	Wright Valley
BT4_17	268	78018	23426	30	350	23265	99.31	BT4	5	Dry	Lake Brownworth	Wright Valley
BT4_9	381	74493	21563	29	350	19910	92.33	BT4	2	Transition	Lake Brownworth	Wright Valley
BT5_0	34	79955	20410	26	350	18982	93.00	BT5	1	Wet	Lake Brownworth	Wright Valley
BT5_11	188	68071	19455	29	350	18697	96.10	BT5	5	Dry	Lake Brownworth	Wright Valley
BT5_2	52	83741	24715	30	350	23621	95.57	BT5	2	Transition	Lake Brownworth	Wright Valley
BT5_5	186	100393	24025	24	350	22968	95.60	BT5	3	Transition	Lake Brownworth	Wright Valley
BT5_6	225	101765	25823	25	350	24260	93.95	BT5	4	Dry	Lake Brownworth	Wright Valley
LB1_0	1040	58481	20693	35	350	20070	96.99	LBo1	1	Wet	Lake Bonney	Taylor Valley
LB1_1	710	88682	35015	39	350	34462	98.42	LBo1	2	Transition	Lake Bonney	Taylor Valley
LB1_6	1	32458	18327	56	350	17934	97.86	LBo1	3	Transition	Lake Bonney	Taylor Valley
LB1_7	23	44974	19124	43	350	18743	98.01	LBo1	4	Dry	Lake Bonney	Taylor Valley
LB1_8	2	78858	43415	55	350	42284	97.39	LBo1	5	Dry	Lake Bonney	Taylor Valley
LB2_0	328	60379	21580	36	350	20521	95.09	LBo2	1	Wet	Lake Bonney	Taylor Valley
LB2_1	338	59666	23457	39	350	23272	99.21	LBo2	2	Transition	Lake Bonney	Taylor Valley
LB2_6	1	6141	3153	51	350	3060	97.05	LBo2	3	Transition	Lake Bonney	Taylor Valley
LB2_8.5	9	7805	4095	52	350	4037	98.58	LBo2	4	Dry	Lake Bonney	Taylor Valley
LB3_0	854	80353	23225	29	350	22466	96.73	LBo3	1	Wet	Lake Bonney	Taylor Valley
LB3_1	366	70635	29784	42	350	29581	99.32	LBo3	2	Transition	Lake Bonney	Taylor Valley
LB3_5.9	1	26788	14393	54	350	14021	97.42	LBo3	3	Transition	Lake Bonney	Taylor Valley
LB3_7	15	57730	24798	43	350	24159	97.42	LBo3	4	Dry	Lake Bonney	Taylor Valley
LB3_8	25	82881	35679	43	350	34207	95.87	LBo3	5	Dry	Lake Bonney	Taylor Valley
LF1_0	1000	78490	27712	35	350	26974	97.34	LF1	1	Wet	Lake Fryxell	Taylor Valley
LF1_1	1080	70882	29129	41	350	28647	98.35	LF1	2	Transition	Lake Fryxell	Taylor Valley
LF1_19	996	48830	23147	47	350	22714	98.13	LF1	5	Dry	Lake Fryxell	Taylor Valley
LF1_7	102	71815	29989	42	350	29688	99.00	LF1	3	Transition	Lake Fryxell	Taylor Valley
LF1_8	400	57759	23345	40	350	23005	98.54	LF1	4	Dry	Lake Fryxell	Taylor Valley
LF2_0	1140	67334	25658	38	350	24617	95.94	LF2	1	Wet	Lake Fryxell	Taylor Valley
LF2_1	1200	35663	16264	46	350	15627	96.08	LF2	2	Transition	Lake Fryxell	Taylor Valley
LF2_10	1120	85438	31455	37	350	31267	99.40	LF2	4	Dry	Lake Fryxell	Taylor Valley
LF2_20.5	722	54496	18718	34	350	18516	98.92	LF2	5	Dry	Lake Fryxell	Taylor Valley
LF2_9	23	77692	28618	37	350	28406	99.26	LF2	3	Transition	Lake Fryxell	Taylor Valley
LF3_0	1160	83743	31708	38	350	30424	95.95	LF3	1	Wet	Lake Fryxell	Taylor Valley
LF3_1	45	62712	23181	37	350	22492	97.03	LF3	2	Transition	Lake Fryxell	Taylor Valley
LF3_20	684	83802	31824	38	350	31228	98.13	LF3	5	Dry	Lake Fryxell	Taylor Valley
LF3_7	181	55089	21624	39	350	21366	98.81	LF3	3	Transition	Lake Fryxell	Taylor Valley
LF3_8.5	836	86817	34639	40	350	34105	98.46	LF3	4	Dry	Lake Fryxell	Taylor Valley
LH1_0	514	73457	27980	38	350	26925	96.23	LH1	1	Wet	Lake Hoare	Taylor Valley
LH1_1	618	76223	26387	35	350	25883	98.09	LH1	2	Transition	Lake Hoare	Taylor Valley
LH1_2	76.4	20742	9738	47	350	9617	98.76	LH1	3	Transition	Lake Hoare	Taylor Valley
LH1_2.5	133.4	57758	22544	39	350	22286	98.86	LH1	4	Dry	Lake Hoare	Taylor Valley
LH1_7	926	94313	34362	36	350	34033	99.04	LH1	5	Dry	Lake Hoare	Taylor Valley
LH2_0	876	71305	27076	38	350	25982	95.96	LH2	1	Wet	Lake Hoare	Taylor Valley
LH2_1	682	89187	33839	38	350	32736	96.74	LH2	2	Transition	Lake Hoare	Taylor Valley
LH2_3	86.4	68540	22653	33	350	22273	98.32	LH2	3	Transition	Lake Hoare	Taylor Valley
LH2_3.5	182.2	74620	26945	36	350	26645	98.89	LH2	4	Dry	Lake Hoare	Taylor Valley
LH2_7	524	57092	18664	33	350	18431	98.75	LH2	5	Dry	Lake Hoare	Taylor Valley
Sp1_0	1040	62267	18894	30	350	17158	90.81	Sp1	1	Wet	Spaulding Pond	Taylor Valley
Sp1_1.5	1080	89714	34991	39	350	32404	92.61	Sp1	2	Transition	Spaulding Pond	Taylor Valley
Sp1_14	111	68442	28012	41	350	27337	97.59	Sp1	5	Dry	Spaulding Pond	Taylor Valley
Sp1_3	824	94708	40310	43	350	39262	97.40	Sp1	3	Transition	Spaulding Pond	Taylor Valley
Sp1_5	1060	87065	37406	43	350	35910	96.00	Sp1	4	Dry	Spaulding Pond	Taylor Valley
Sp3_0	888	137905	52784	38	350	46087	87.31	Sp3	1	Wet	Spaulding Pond	Taylor Valley
Sp3_1	822	98758	36016	36	350	31029	86.15	Sp3	2	Transition	Spaulding Pond	Taylor Valley
Sp3_16	1000	72169	28614	40	350	27871	97.40	Sp3	5	Dry	Spaulding Pond	Taylor Valley
Sp3_3	952	92425	37294	40	350	35588	95.43	Sp3	3	Transition	Spaulding Pond	Taylor Valley
Sp3_6	640	48311	20398	42	350	19830	97.22	Sp3	4	Dry	Spaulding Pond	Taylor Valley
Sp4_0	1020	67494	24497	36	350	21489	87.72	Sp4	1	Wet	Spaulding Pond	Taylor Valley
Sp4_10	982	88463	30574	35	350	29514	96.53	Sp4	4	Dry	Spaulding Pond	Taylor Valley
Sp4_14	990	72375	25536	35	350	25093	98.27	Sp4	5	Dry	Spaulding Pond	Taylor Valley
Sp4_3	992	78510	29776	38	350	27872	93.61	Sp4	2	Transition	Spaulding Pond	Taylor Valley
Sp4_5	950	79454	28219	36	350	25979	92.06	Sp4	3	Transition	Spaulding Pond	Taylor Valley
VT4_1	19	65763	17173	26	350	16892	98.36	VT4	1	Wet	Lake Vanda	Wright Valley
VT4_10	141	59432	19335	33	350	19166	99.13	VT4	5	Dry	Lake Vanda	Wright Valley
VT4_2	17	83147	29868	36	350	29434	98.55	VT4	2	Transition	Lake Vanda	Wright Valley
VT4_3.3	19	80386	24525	31	350	24089	98.22	VT4	3	Transition	Lake Vanda	Wright Valley
VT4_4	31	81458	21858	27	350	21660	99.09	VT4	4	Dry	Lake Vanda	Wright Valley
VT6_1	30	65536	19713	30	350	19470	98.77	VT6	1	Wet	Lake Vanda	Wright Valley
VT6_2	17	121604	57601	47	350	57262	99.41	VT6	2	Transition	Lake Vanda	Wright Valley
VT6_3.5	19	39133	14230	36	350	13975	98.21	VT6	3	Transition	Lake Vanda	Wright Valley
VT6_4.5	31	71480	23208	32	350	23132	99.67	VT6	4	Dry	Lake Vanda	Wright Valley
VT6_9	58	293	*all filtered out*	N/A	N/A	N/A	N/A	VT6	5	Dry	Lake Vanda	Wright Valley
VT7_1	92	50732	15103	30	350	14858	98.38	VT7	1	Wet	Lake Vanda	Wright Valley
VT7_12	40	53999	17835	33	350	17669	99.07	VT7	5	Dry	Lake Vanda	Wright Valley
VT7_3	5	72277	25442	35	350	25009	98.30	VT7	2	Transition	Lake Vanda	Wright Valley
VT7_4.5	27	79156	25321	32	350	24945	98.52	VT7	3	Transition	Lake Vanda	Wright Valley
VT7_6	15	51649	18937	37	350	18644	98.45	VT7	4	Dry	Lake Vanda	Wright Valley

Table S2 - Tukey multiple comparisons of means for beta-dispersion (betadisp) results between the three different zones for each lake transect.

**Lake Fryxell**	**p-value**
Transition-Dry	<0.01
Wet-Dry	0.44
Wet- Transition	<0.001
**Lake Bonney**	**p-value**
Transition -Dry	0.02
Wet-Dry	0.70
Wet- Transition	0.01
**Lake Hoare**	**p-value**
Transition -Dry	0.11
Wet-Dry	0.89
Wet- Transition	0.36
**Sapulding Pond**	**p-value**
Transition -Dry	0.06
Wet-Dry	0.43
Wet- Transition	0.68
**Lake Vanda**	**p-value**
Transition -Dry	<0.01
Wet-Dry	0.74
Wet- Transition	<0.01
**Lake Brownworth**	**p-value**
Transition -Dry	0.94
Wet-Dry	0.57
Wet- Transition	0.71

Table S3 – Top twenty bacterial OTUs classified by Random Forest analysis as the most important to discriminate between the different zones of lake transects. The importance of OTUs is determined by the Mean Decrease in Accuracy from permuting the values in each feature. Mean Decrease in Accuracy contains a measure of the extent to which a variable improves the accuracy of the forest in predicting the classification.

OTU ID	Kingdom	Phylum	Class	Order	Family	Genus	Specie
OTU312	Bacteria	Bacteroidota	Bacteroidia	Flavobacteriales	Flavobacteriaceae	Flavobacterium	Unclassified
OTU6	Bacteria	Bacteroidota	Bacteroidia	Chitinophagales	Chitinophagaceae	Segetibacter	Unclassified
OTU40	Bacteria	Bacteroidota	Bacteroidia	Chitinophagales	Chitinophagaceae	Ferruginibacter	Unclassified
OTU93	Bacteria	Verrucomicrobiota	Verrucomicro	Verrucomicrobiales	Rubritaleaceae	Luteolibacter	Unclassified
OTU150	Bacteria	Chloroflexi	Chloroflexia	Kallotenuales	AKIW781	Unclassified	Unclassified
OTU27	Bacteria	Deinococcota	Deinococci	Deinococcales	Trueperaceae	Truepera	Unclassified
OTU164	Bacteria	Abditibacteriota	Abditibacteri	Abditibacteriales	Abditibacteriaceae	Abditibacterium	Unclassified
OTU280	Bacteria	Abditibacteriota	Abditibacteri	Abditibacteriales	Abditibacteriaceae	Abditibacterium	Unclassified
OTU72	Bacteria	Abditibacteriota	Abditibacteri	Abditibacteriales	Abditibacteriaceae	Abditibacterium	Unclassified
OTU25	Bacteria	Acidobacteriota	Blastocatelli	Blastocatellales	Blastocatellaceae	Blastocatella	Unclassified
OTU9	Bacteria	Acidobacteriota	Blastocatelli	Blastocatellales	Blastocatellaceae	Blastocatella	Unclassified
OTU31	Bacteria	Proteobacteria	Gammaprote	Xanthomonadales	Xanthomonadaceae	Pseudoxanthomonas	Unclassified
OTU44	Bacteria	Proteobacteria	Gammaprote	Xanthomonadales	Xanthomonadaceae	Thermomonas	Unclassified
OTU126	Bacteria	Proteobacteria	Alphaproteol	Sphingomonadales	Sphingomonadaceae	Sphingorhabdus	Unclassified
OTU33	Bacteria	Proteobacteria	Alphaproteol	Sphingomonadales	Sphingomonadaceae	Sphingomonas	Unclassified
OTU1839	Bacteria	Proteobacteria	Alphaproteol	Caulobacterales	Caulobacteraceae	Brevundimonas	Unclassified
OTU73	Bacteria	Actinobacteriota	Rubrobacteri	Rubrobacterales	Rubrobacteriaceae	Rubrobacter	Unclassified
OTU39	Bacteria	Actinobacteriota	Thermoleoph	Solirubrobacterales	Solirubrobacteraceae	Unclassified	Unclassified
OTU134	Bacteria	Actinobacteriota	Thermoleoph	Solirubrobacterales	67-14	Unclassified	Unclassified
OTU2208	Bacteria	Actinobacteriota	Actinobacter	Propionibacteriales	Nocardioidaceae	Marmoricola	Unclassified
